# Standardized *Centella asiatica* (ECa 233) extract decreased pain hypersensitivity development in a male mouse model of chronic inflammatory temporomandibular disorder

**DOI:** 10.1038/s41598-023-33769-w

**Published:** 2023-04-24

**Authors:** Nattapon Rotpenpian, Aree Wanasuntronwong, Sompol Tapechum, Anchalee Vattarakorn, Chit Care, Wongsathit Chindasri, Kanokwan Tilokskulchai, Mayuree H. Tantisira, Narawut Pakaprot

**Affiliations:** 1 Department of Physiology, Faculty of Medicine Siriraj Hospital, Mahidol University, 2 Srisavarindhira Bldg., 13Th Floor, Wanglang Road, Siriraj Subdistrict, Bangkoknoi District, Bangkok, 10700 Thailand; 2 Department of Oral Biology and Occlusion, Faculty of Dentistry, Prince of Songkla University, Songkhla, Thailand; 3 Department of Oral Biology, Faculty of Dentistry, Mahidol University, Bangkok, Thailand; 4 Faculty of Pharmaceutical Sciences, Burapha University, Chonburi, Thailand

**Keywords:** Drug discovery, Neuroscience, Somatosensory system, Pain, Chronic pain, Physiology, Neurophysiology, Experimental models of disease, Medical research, Drug development, Neurology, Neurological disorders

## Abstract

Chronic inflammatory temporomandibular disorder (TMD) pain has a high prevalence, and available nonspecific treatments have adverse side effects. ECa 233, a standardized *Centella asiatica* extract, is highly anti-inflammatory and safe. We investigated its therapeutic effects by injecting complete Freund’s adjuvant (CFA) into right temporomandibular joint of mice and administering either ibuprofen or ECa 233 (30, 100, and 300 mg/kg) for 28 days. Inflammatory and nociceptive markers, bone density, and pain hypersensitivity were examined. CFA decreased ipsilateral bone density, suggesting inflammation localization, which ipsilaterally caused immediate calcitonin gene-related peptide elevation in the trigeminal ganglia (TG) and trigeminal subnucleus caudalis (TNC), followed by late increase of NaV1.7 in TG and of p-CREB and activation of microglia in TNC. Contralaterally, only p-CREB and activated microglia in TNC showed delayed increase. Pain hypersensitivity, which developed early ipsilaterally, but late contralaterally, was reduced by ibuprofen and ECa 233 (30 or 100 mg/kg). However, ibuprofen and only 100-mg/kg ECa 233 effectively mitigated marker elevation. This suggests 30-mg/kg ECa 233 was antinociceptive, whereas 100-mg/kg ECa 233 was both anti-inflammatory and antinociceptive. ECa 233 may be alternatively and safely used for treating chronic inflammatory TMD pain, showing an inverted U-shaped dose–response relationship with maximal effect at 100 mg/kg.

## Introduction

Orofacial pain is the most prevalent and debilitating condition affecting the craniofacial region, accounting for approximately 10–15% of all pains^1^. Temporomandibular disorder (TMD) pain is a major pathological condition (roughly 15–20% of the orofacial pain) causing widespread chronic pain in the orofacial area^2^. The trigeminal pain pathway is linked to orofacial pain development. Pain signals travel through the peripheral processes of the first-order sensory neurons in the trigeminal ganglia (TG) in the peripheral nervous system (PNS) and are conveyed through their central processes to the second-order sensory neurons in the trigeminal subnucleus caudalis (TNC) in the central nervous system (CNS). In acute inflammatory orofacial pain, TG neurons primarily release calcitonin gene-related peptide (CGRP)—an inflammatory mediator and a neurogenic inflammatory marker—into TG and TNC after excessive uncontrolled inflammation. In chronic pain, the released CGRP in TG is implicated in the increased expression of voltage-gated sodium ion channel 1.7 (NaV1.7)—a pain hypersensitivity marker in PNS—causing peripheral sensitization^3^. The released CGRP in TNC is also associated with enhanced expression of activated microglia and phosphorylated cAMP response element-binding protein (p-CREB)—a marker for nociceptive activity and pain hypersensitivity in CNS—causing central sensitization^4^. This proposed mechanism results in development of orofacial pains, including TMD pain.

TMD pain results from multiple factors affecting the temporomandibular joint (TMJ) such as trauma or occlusal changes^5^. Because the pathophysiology of chronic TMD pain remains unclear, it is typically managed by nonspecific treatments, such as nonsteroidal anti-inflammatory drugs (NSAIDs), resulting in unpredictable outcomes and/or adverse effects^6^. Although widely used, NSAIDs produce many known adverse effects, including gastrointestinal irritation and kidney injury^7^. Thus, new treatment innovations particularly focus on the use of traditional herbal medicines that may treat chronic inflammatory TMD pain without adverse side effects. The herbal plant *Centella asiatica* (L.) Urban (Indian pennywort, Gotu kola, or Bua-bok) reportedly exhibits anti-inflammatory and antinociceptive effects^8^. Its major bioactive constituents are triterpenoid glycosides including asiaticoside and madecassoside^8,9^. However, its effects remain inconsistent^9^. This inconsistency could be explained by the greatly varying amounts of the herb’s active components, depending on many factors such as cultivation methods and climate. Hence, researchers established a standardized *C. asiatica* extract through guided isolation by controlling the manufacturing process; subsequently, the standardized extract of *C. asiatica*, namely, ECa 233, was obtained. ECa 233 is a white/off-white powder with ≥ 80% w/w of triterpenoid glycosides, with a consistent madecassoside-to-asiaticoside ratio of 1.5 ± 0.5:1^9^.

Both acute and subchronic toxicity tests revealed that ECa 233 is very safe to use^10^. ECa 233 did not induce nor inhibit the activities of drug-metabolizing enzymes, which eliminate and/or detoxify major drug groups such as paracetamol and NSAIDs; hence, ECa 233 does not interfere with major drug metabolisms^9,10^. Moreover, ECa 233 has neuropharmacological activities such as anti-inflammation and antinociception. Its antinociceptive activity was explored in a tail flick test, showing that ECa 233 reduced pain responses^11^. In patients with aphthous ulcers, an oral paste containing ECa 233 considerably reduced ulcer-associated pain and inflammation^12^. These effects might occur through the antinociceptive and anti-inflammatory mechanisms of ECa 233. Further, some studies have investigated the anti-inflammatory activities of *C. asiatica* extract in acute inflammatory pain such as in osteoarthritis of knee joints^13^. Recently, our group reported the anti-inflammatory activities of *C. asiatica* extract in a mouse model of acute temporomandibular joint osteoarthritis in which ECa 233 could inhibit pathogenesis in the affected joint by modulating the local expression of transient receptor potential vanilloid 1 (TRPV1) and acid-sensing ion channel subunit 3 (ASIC3)^14^.

Although ECa 233 has beneficial effects, its underlying mechanisms remain poorly explored. Therefore, we aimed to elucidate its effects and underlying mechanisms on chronic inflammatory TMD pain induced by unilaterally injecting complete Freund’s adjuvant (CFA) into the right TMJ of mice. Our study might be the first to demonstrate the benefits of *C. asiatica* extract on chronic inflammatory TMD pain.

## Results

### Descriptive results

#### Weight

**Figure 1:**
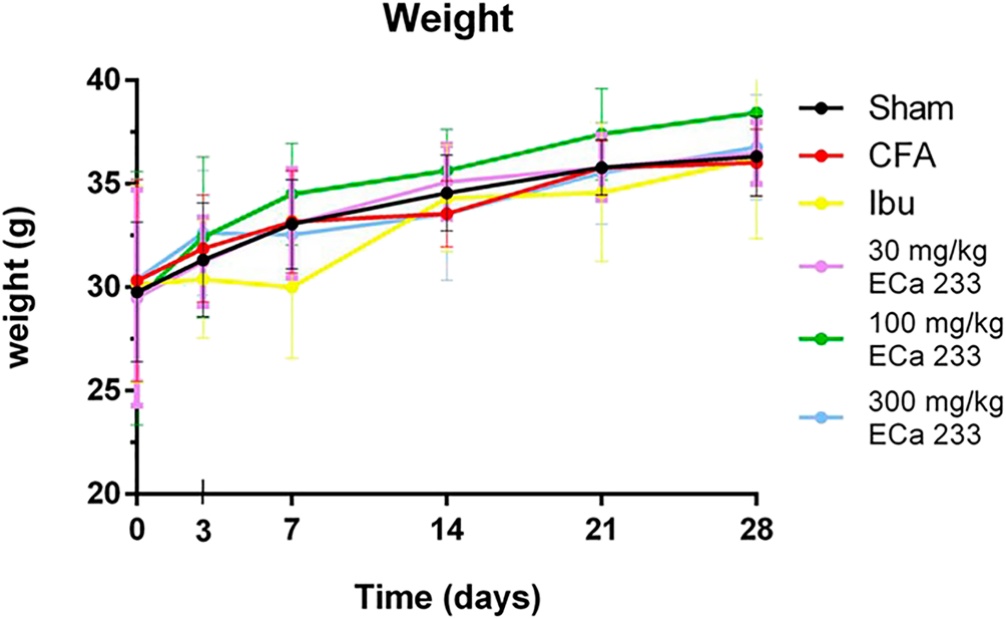
No difference in weight in all groups at every time point (mean ± SEM; n = 9, two-way analysis of variance). CFA, complete Freund’s adjuvant; Ibu, ibuprofen.

After 28 days of CFA-induced TMD pain, there was no difference in weight between the Sham, Ibu, CFA, 30ECa, 100ECa, and 300ECa groups (Fig. 1).

### Orofacial pain sensitivity

**Figure 2:**
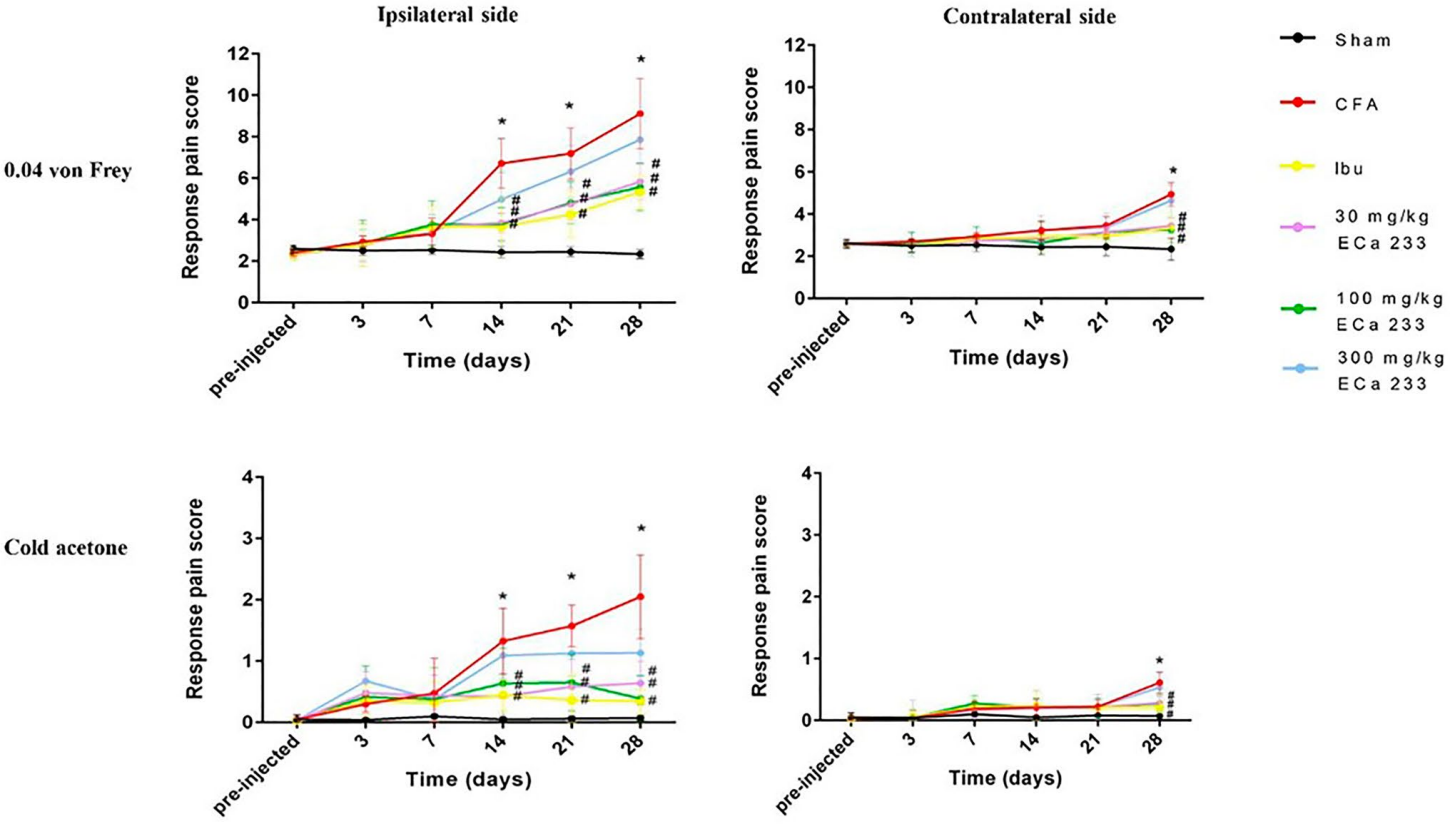
Pain response scores tested by von Frey filament and cold acetone tests performed at different time points in all groups. In the complete Freund’s adjuvant (CFA) group, the pain scores of all behavioral tests significantly increased on the ipsilateral side at post–CFA-injection days 14 [F(5,48) = 16.187, P < 0.05] and 21 [F(5,48) = 15.159, P < 0.05], and 28 [F(5,48) = 18.299, P < 0.05] as per von-Frey test and at days 14 [F(5,48) = 11.367, P < 0.05], 21 [F(5,48) = 12.357, P < 0.05] and 28 [F(5,48) = 17.045, P < 0.05] as per cold acetone test, and on the contralateral side at post-CFA-injection day 28 [F(5,48) = 19.543, P < 0.05, F(5,48) = 17.697, P < 0.05, von-Frey test and cold acetone test, respectively]. In the Ibu, 30ECa, and 100ECa groups, the pain scores significantly decreased on the ipsilateral side, but not in the 300ECa group, at post–CFA-injection days 14, 21, and 28 {as per the von-Frey test, Ibu: [F(5,48) = 17.299, P < 0.05], [F(5,48) = 16.199, P < 0.05], and [F(5,48) = 17.299, P < 0.05]; 30ECa: [F(5,48) = 16.215, P < 0.05], [F(5,48) = 16.058, P < 0.05], and [F(5,48) = 16.192, P < 0.05]; 100ECa: [F(5,48) = 18.252, P < 0.05], [F(5,48) = 16.358, P < 0.05], and [F(5,48) = 16.014, P < 0.05]; and 300ECa: [F(5,48) = 28.512, P = 0.159], [F(5,48) = 26.358, P = 0.125], and [F(5,48) = 26.514, P = 0.201] and as per the cold acetone test, Ibu: [F(5,48) = 15.211, P < 0.05], [F(5,48) = 14.992, P < 0.05], and [F(5,48) = 16.129, P < 0.05]; 30ECa: [F(5,48) = 16.058, P < 0.05], [F(5,48) = 15.018, P < 0.05], and [F(5,48) = 16.047, P < 0.05]; 100ECa: [F(5,48) = 17.931, P < 0.05], [F(5,48) = 16.308, P < 0.05], and [F(5,48) = 16.703, P < 0.05]; and 300ECa: [F(5,48) = 21.434, P = 0.149], [F(5,48) = 25.384, P = 0.135], and [F(5,48) = 26.014, P = 0.230]}. On the contralateral side, the pain scores significantly decreased in the Ibu, 30ECa, and 100ECa groups at post–CFA-injection day 28 for von-Frey test (Ibu: [F (5,48) = 11.251, P < 0.05]; 30ECa: [F(5,48) = 10.267, P < 0.05]; 100ECa: [F(5,48) = 11.004, P < 0.05]) for cold acetone test (Ibu: [F(5,48) = 10.501, P < 0.05]; 30ECa: [F(5,48) = 10.707, P < 0.05]; 100ECa: [F(5,48) = 10.804, P < 0.05]) compared with that in the CFA group. (mean ± SEM; n = 9; *P < 0.05 compared with the Sham group, ^#^P < 0.05 compared with the CFA group, two-way analysis of variance followed by Fisher’s least significant difference post hoc test).

Figure 2 illustrates the time course of behavioral pain responses of the different groups tested by 0.04 g von Frey filament for mechanical hyperalgesia and 0.05 ml cold acetone for cold allodynia. We tested each side 12 times and then recorded the response scores according to the following criteria: no response = 0, occurrence of head withdrawal = 0.25, occurrence of face grooming once = 1, and occurrence of face grooming > 3 times = 1.5. We added all response scores on each side to obtain the total response score; the maximum response score was 18. The Sham group, which was injected with NSS into the right TMJ, showed the lowest pain response scores; the response remained constant at the baseline level throughout the whole experiment. However, the CFA group developed a significant pain hypersensitivity on the ipsilateral side of the CFA injection at post-CFA-injection days 14, 21, and 28 with both von-Frey and cold acetone tests, and on the contralateral side at post-CFA-injection day 28 also with both von-Frey and cold acetone tests when compared with the Sham group. In the Ibu, 30ECa, and 100ECa groups, pain hypersensitivity significantly decreased on the ipsilateral side, but not in the 300ECa group, at post–CFA-injection days 14, 21, and 28 with both von-Frey and cold acetone tests. On the contralateral side, pain hypersensitivity significantly decreased in the Ibu, 30ECa, and 100ECa groups at post–CFA-injection day 28 for both von-Frey and cold acetone tests, compared with that in the CFA group. Thus, CFA injection induced pain hypersensitivity, including mechanical hyperalgesia and cold allodynia, initially on the ipsilateral side of the injection and later on the contralateral side. In addition, ibuprofen, which was used as a positive control treatment, and ECa 233 at 30 and 100 mg/kg doses significantly and equally reduced pain hypersensitivity that developed bilaterally, while there was no statistical difference among the three groups at any days measured.

### Structural changes in TMJ

**Figure 3:**
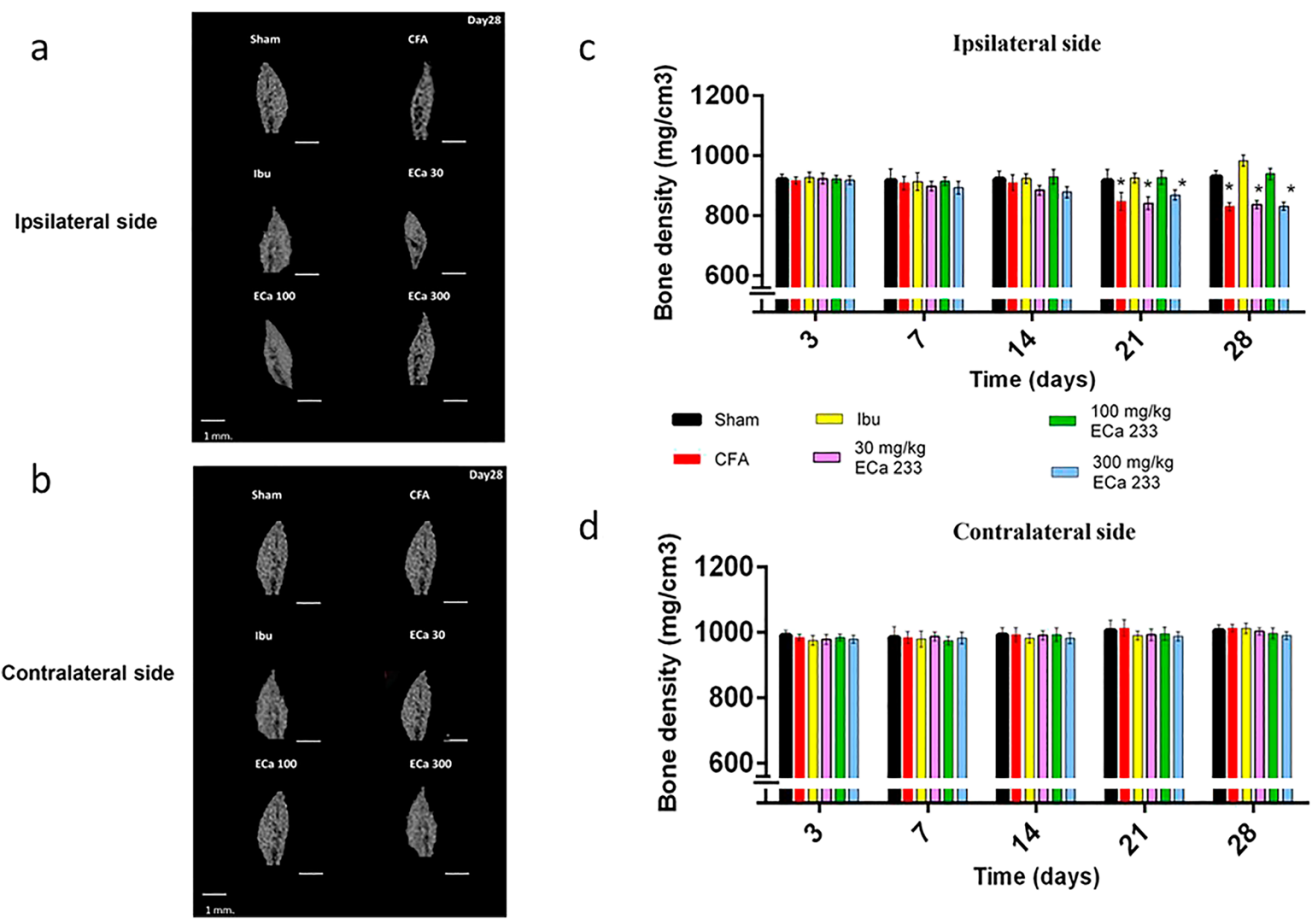
Bone density changes in the condylar head of temporomandibular joints (TMJs) determined by micro–computed tomography (micro-CT). (**a**,**b**) Sample images of micro-CT scans of the ipsilateral and contralateral TMJs at post–CFA-injection day 28, scale bar = 1 mm. (**c**) Bone density changes on the ipsilateral side of the TMJ’s condylar head. The groups treated with complete Freund’s adjuvant, 30 mg/kg ECa 233, and 300 mg/kg ECa 233 (CFA, 30ECa, and 300ECa groups, respectively) had significant bone density reductions on the ipsilateral side at post–CFA-injection days 21 (CFA [F(5,48) = 60.339, P < 0.05], 30ECa [F(5,48) = 58.931, P < 0.05], 300ECa [(5,48) = 61.135, P < 0.05]) and 28 (CFA [F(5,48) = 49.158, P < 0.05], 30ECa [(5,48) = 48.621, P < 0.05], 300ECa [(5,48) = 47.996, P < 0.05]) compared with the Sham group whereas the Ibu and 100ECa groups did not showed bone density reduction on both days (day 21: Ibu [F(5,48) = 77.678, P = 0.324], 100ECa [F(5,48) = 87.678, P = 0.424]; day 28: Ibu [F(5,48) = 78.728, P = 0.334], 100ECa [F(5,48) = 74.018, P = 0.384]) (mean ± SEM; n = 3/group/time point; *P < 0.05, two-way analysis of variance followed by Fisher’s least significant difference post hoc test). (**d**) Bone density changes on the contralateral side of the TMJ’s condylar head. No difference was observed in bone density on the contralateral side in all groups. (mean ± SEM; n = 3/group/time point).

Figure 3 depicts the bone density of the condylar head of TMJ on the ipsilateral and contralateral sides measured by micro-CT. Although CFA-induced pain hypersensitivity developed early on the ipsilateral side and later on the contralateral side, the bone density of the CFA group significantly reduced only on the ipsilateral side at post–CFA-injection days 21 and 28, compared with that of the Sham group. Considering that bone density reduction is a major sign of TMJ tissue inflammation, CFA-induced inflammation could cause pathology in the condylar head of the right TMJ, consistent with pain hypersensitivity on the ipsilateral side. Meanwhile, the pain hypersensitivity on the contralateral side seemed not to be induced by the pathology in the contralateral TMJ because no bone density reduction was observed. Compared with the Sham group, only the Ibu and 100ECa groups did not exhibit bone density reduction at post–CFA-injection days 21 and 28. Pain hypersensitivity was also reduced in the 30ECa group; however, this group as well as the CFA and 300ECa groups showed bone density reduction without significant difference among the three groups. Thus, both 30 and 300 mg/kg doses of ECa 233 could not prevent the pathology caused by CFA, whereas 0.14 g/kg ibuprofen and 100 mg/kg ECa 233 effectively prevented the pathology at all of the time points after CFA injection and also reduced pain hypersensitivity.

### Inflammation and nociceptive activity changes in TG

#### Expression of CGRP in TG

**Figure 4:**
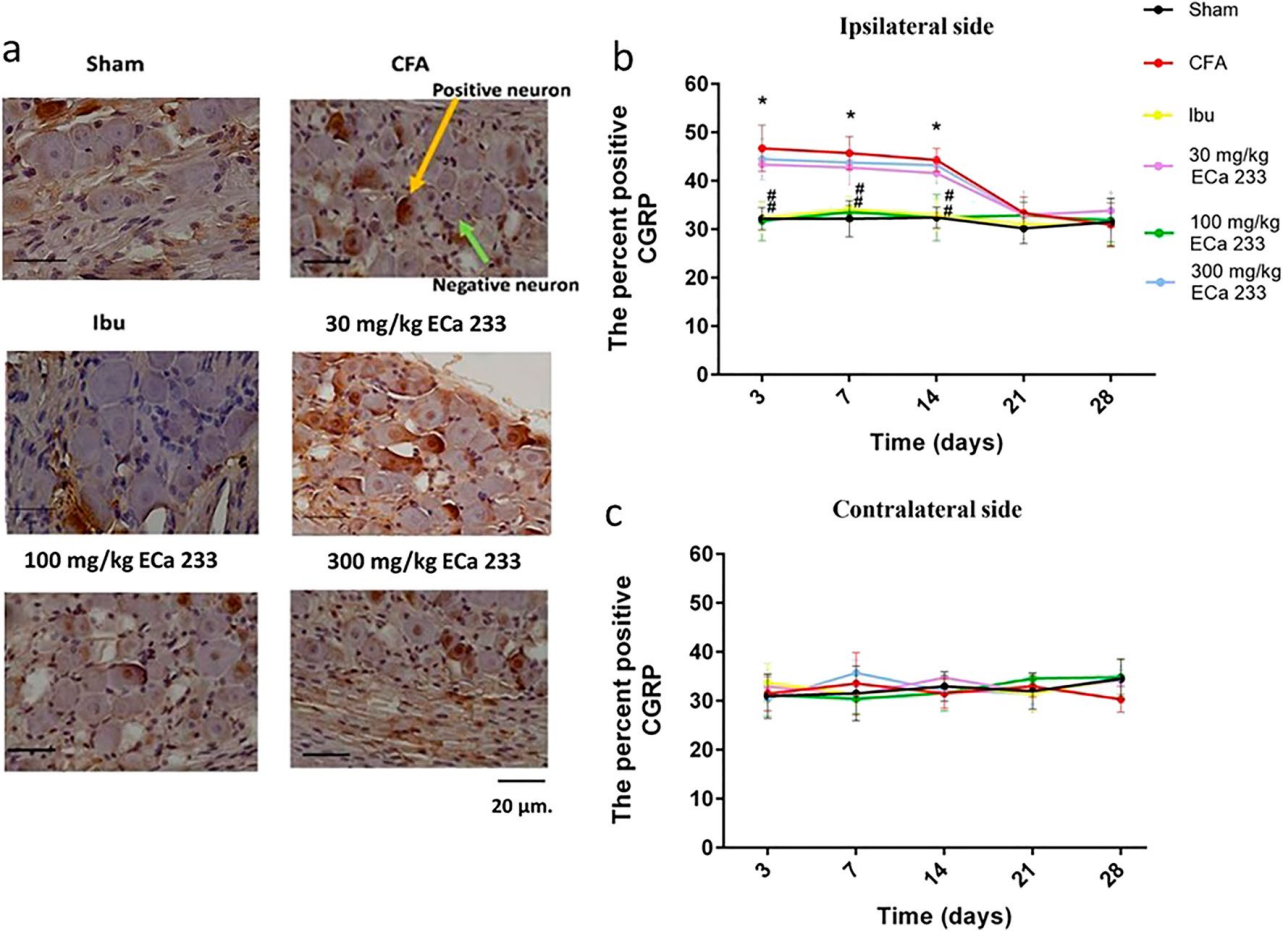
Calcitonin gene-related peptide (CGRP) expression in TGs in all groups at different time points. (**a**) An example of CGRP staining by immunohistochemistry at the ipsilateral trigeminal ganglia (TG) at post–CFA-injection day 3, scale bar = 20 µm. (**b**,**c**) Percentage of positive CGRP neurons per the total number of neurons in different groups. The CGRP expression in the complete Freund’s adjuvant–treated (CFA) group increased in the ipsilateral TG at post–CFA-injection days 3 [F(5,48) = 17.046, P < 0.05], 7 [F(5,48) = 16.046, P < 0.05], and 14 [F(5,48) = 15.048, P < 0.05] compared with that in the Sham group. Likewise, the CGRP expression in the groups treated with ibuprofen and 100 mg/kg ECa 233 (Ibu and 100ECa groups, respectively) significantly decreased in the ipsilateral TG at post–CFA-injection days 3 (Ibu [F(5,48) = 16.541, P < 0.05], 100ECa [F(5,48) = 17.291, P < 0.05]), 7 (Ibu [F(5,48) = 13.421, P < 0.05], 100ECa [F(5,53) = 14.913, P < 0.05]), and 14 (Ibu [F(5,48) = 13.421, P < 0.05], 100ECa [F(5,53) = 15.931, P < 0.05]), compared with that in the CFA group. In contrast to Ibu and 100ECa groups, the 30ECa and 300ECa groups could not show the decreased CGRP expression (day 3: 30ECa [F(5,48) = 33.541, P = 0.254], 300ECa [F(5,48) = 40.129, P = 0.354]; day 7: 30ECa [F(5,48) = 32.134, P = 0.265], 300ECa [F(5,48) = 41.229, P = 0.334]; day 14: 30ECa [F(5,48) = 31.334, P = 0.255], 300ECa [F(5,48) = 40.091, P = 0.374]). In the contralateral TG, the CGRP expression did not change by CFA on any days (day 3: [F(5,48) = 31.254, P = 0.220]; day 7: [F(5,48) = 30.324, P = 0.213]; day 14: [F(5,48) = 33.424, P = 0.296]). (mean ± SEM; n = 3/group/time point; *P < 0.05 compared with the Sham group, ^#^P < 0.05 compared with the CFA group, two-way analysis of variance followed by Fisher’s least significant difference post hoc test).

Figures 4 and 9 illustrate the expression of CGRP in bilateral TGs. CFA injection immediately and significantly enhanced the CGRP expression in the ipsilateral TG at post–CFA-injection days 3, 7, and 14, but not in the contralateral TG, compared with Sham. These data supported the idea that CFA-induced inflammation on the ipsilateral side immediately activates nociceptors, possibly causing pain hypersensitivity on the same side. Although the 30 and 300 mg/kg doses of ECa 233 could not reduce the enhanced expression of CGRP, ibuprofen and 100 mg/kg ECa 233 effectively prevented the enhanced CGRP expression at post–CFA-injection days 3, 7, and 14 compared with the CFA injection. Thus, 100 mg/kg dose of ECa 233 effectively inhibited inflammation in TG.

#### Expression of NaV1.7 in TG

**Figure 5:**
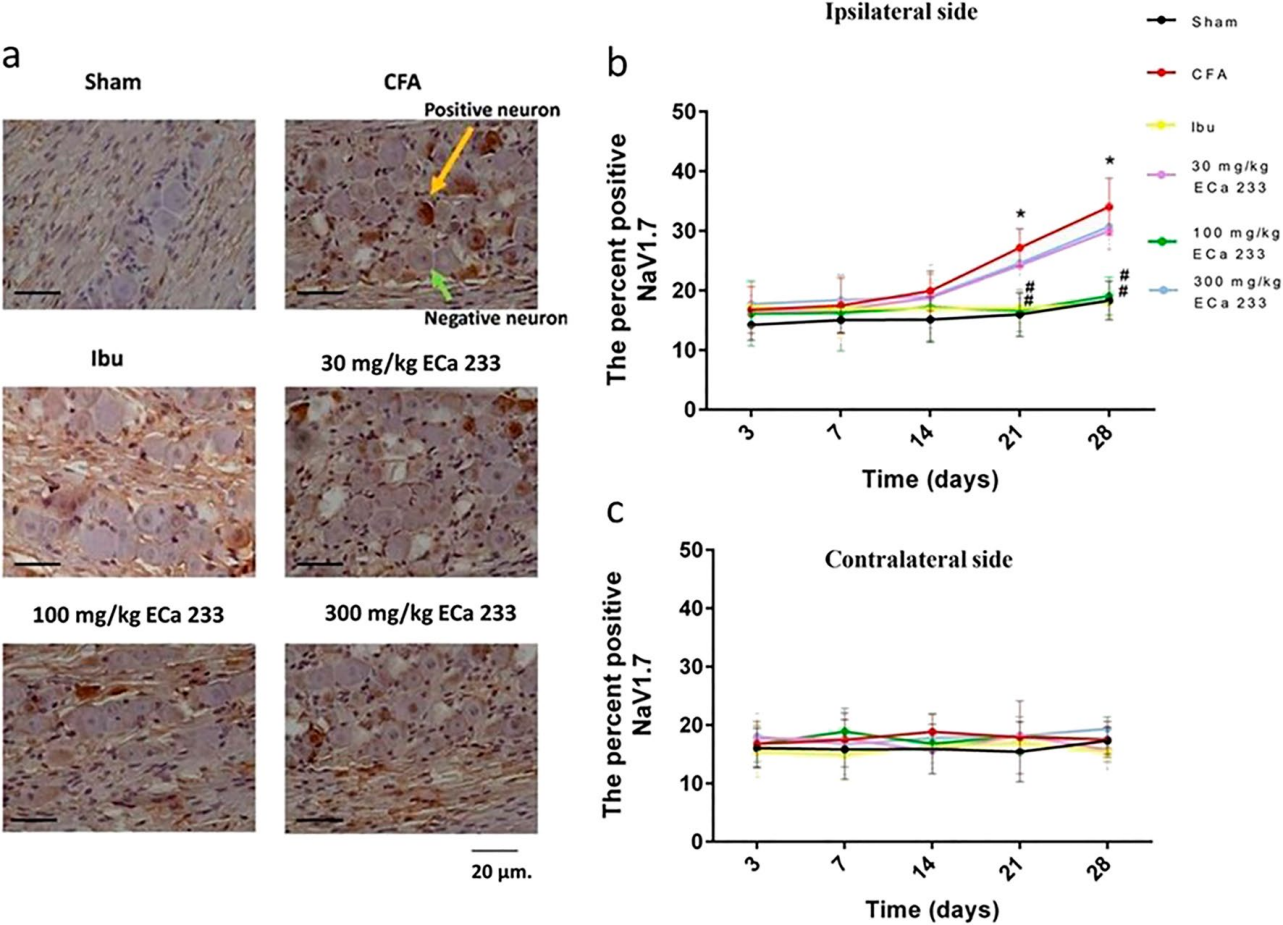
Voltage-gated sodium ion channel 1.7 (NaV1.7) expression in TGs in all groups at different time points. (**a**) An example of NaV1.7 staining by immunohistochemistry at the ipsilateral TG at post–CFA-injection day 28, scale bar = 20 µm. (**b**,**c**) Percentage of positive NaV1.7 neurons per total number of neurons in different groups. At post–CFA-injection days 21 and 28 in the ipsilateral TG, the NaV1.7 expression in the complete Freund’s adjuvant–treated (CFA) group increased compared with that in the Sham group (day 21 [F(5,48) = 9.463, P < 0.05], day 28 [F(5,48) = 8.633, P < 0.05]), whereas that in the groups treated with ibuprofen and 100 mg/kg ECa 233 (Ibu and 100ECa groups, respectively) significantly decreased (day 21: Ibu [F(5,48) = 10.521, P < 0.05], 100ECa [F(5,48) = 12.545, P < 0.05]; day 28: Ibu [F(5,48) = 9.452, P < 0.05], 100ECa [F(5,48) = 10.985, P < 0.05]) compared with that in the CFA group. Both 30ECa and 300ECa showed no difference from the CFA group (day 21: 30ECa [F(5,48) = 5.9372, P = 0.183], 300ECa [F(5,48) = 6.937, P = 0.192]; day 28: 30ECa [F(5,48) = 5.342, P = 0.173], 300ECa [F(5,48) = 6.937, P = 0.192]). In the contralateral TG, CFA did not change the NaV1.7 expression (day 21 [F(5,48) = 1.925, P = 0.132], day 28 [F(5,48) = 1.895, P = 0.113]) (mean ± SEM; n = 3/groups at each time point; *P < 0.05 compared with the Sham group, ^#^P < 0.05 compared with the CFA group, two-way analysis of variance followed by Fisher’s least significant difference post hoc test).

Figures 5 and 9 show the NaV1.7 expression in bilateral TGs. In the CFA group, NaV1.7 expression was late yet significantly enhanced in the ipsilateral TG at post–CFA-injection days 21 and 28, but not in the contralateral TG. The immediate enhancement of CGRP and later enhancement of NaV1.7 corresponded well with the early development of pain hypersensitivity on the ipsilateral side. However, the late development of pain hypersensitivity on the contralateral side seemed to be not associated with the contralateral TG activity because no inflammation and nociceptive activities were observed. Although the 30 and 300 mg/kg doses of ECa 233 could not reduce the enhanced expression of NaV1.7, ibuprofen and 100 mg/kg ECa 233 effectively prevented the enhanced NaV1.7 expression compared with the CFA injection. Thus, 100 mg/kg dose of ECa 233 effectively prevented peripheral sensitization and pain hypersensitivity in TG.

### Inflammation and nociceptive activity changes in TNC

#### Expression of CGRP in TNC

**Figure 6:**
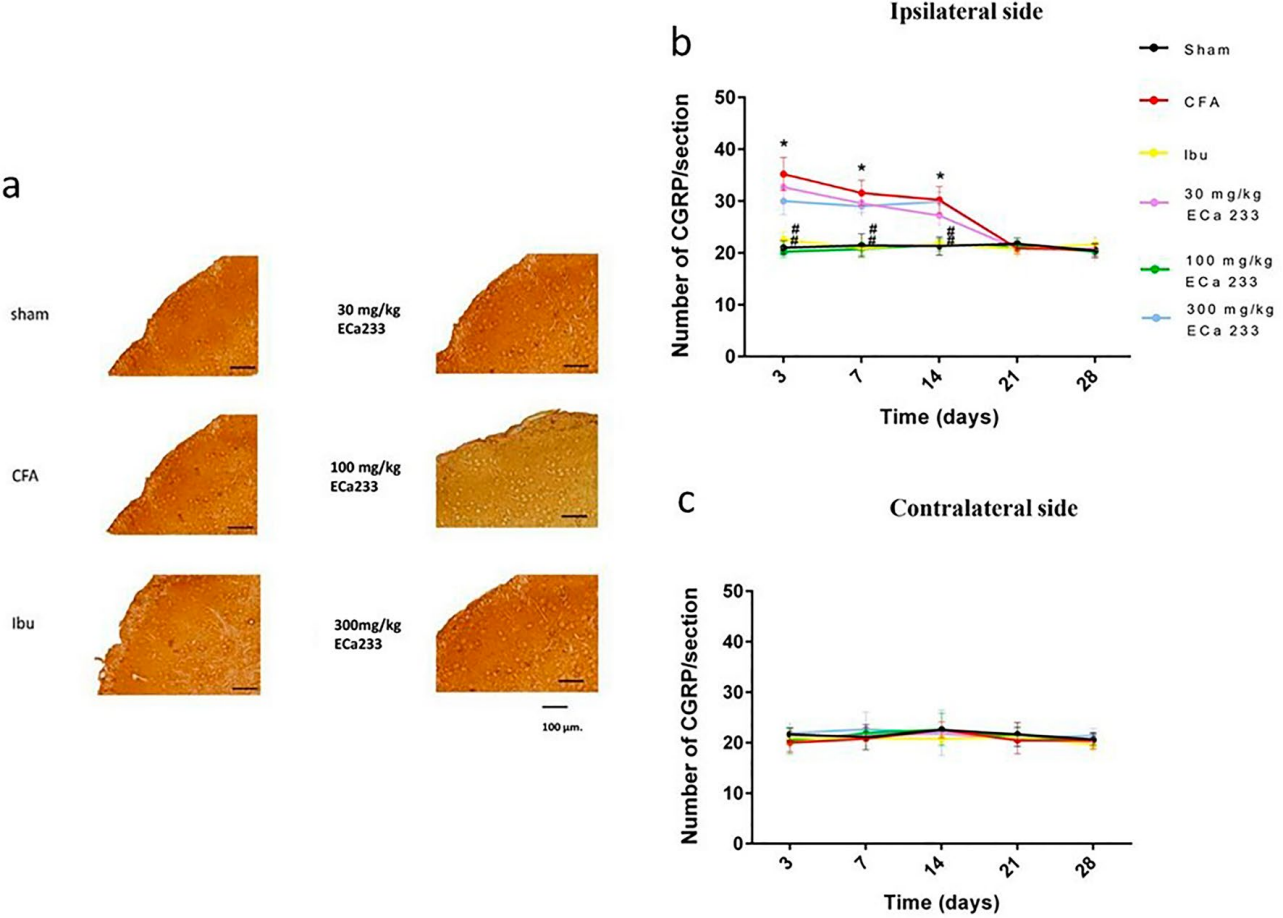
Calcitonin gene-related peptide (CGRP) expression in the trigeminal subnucleus caudalis (TNC) in all groups at different time points. (**a**) An example of CGRP staining by immunohistochemistry at the ipsilateral TNC at post–CFA-injection day 3, scale bar = 100 µm. (**b**,**c**) Number of positive CGRP expression in both sides of TNC in different groups. At post–CFA-injection days 3, 7, and 14 in the ipsilateral TNC, the CGRP expression in the complete Freund’s adjuvant–treated (CFA) group increased compared with that in the Sham group (day 3 [F(5,48) = 13.106, P < 0.05], day 7 [F(5,48) = 15.826, P < 0.05], day14 [F(5,48) = 14.806, P < 0.05]), whereas that in the groups treated with ibuprofen and 100 mg/kg ECa 233 (Ibu and 100ECa groups, respectively) significantly decreased compared with that in the CFA group (day 3: Ibu [F(5,48) = 10.452, P < 0.05], 100ECa [F(5,48) = 12.885, P < 0.05]; day 7: Ibu [F(5,48) = 12.342, P < 0.05], 100ECa [F(5,48) = 12.185, P < 0.05]; day 14: Ibu [F(5,48) = 13.452, P < 0.05], 100ECa [F(5,48) = 12.235, P < 0.05]) Both 30ECa and 300ECa showed no difference from the CFA group (day 3: 30ECa [F(5,48) = 21.485, P = 0.221], 300ECa [F(5,48) = 22.485, P = 0.281]; day 7: 30ECa [F(5,48) = 22.545, P = 0.261], 300ECa [F(5,48) = 21.345, P = 0.293]; day 14: 30ECa [F(5,48) = 20.698, P = 0.231], 300ECa [F(5,48) = 20.435, P = 0.267]). In the contralateral TNC, CFA injection did not change the CGRP expression (day 3 [F(5,48) = 19.944, P = 0.363], day 7 [F(5,48) = 17.444, P = 0.263], day 14 [F(5,48) = 20.124, P = 0.353]) (mean ± SEM; n = 3/group/time point; *P < 0.05 compared with the Sham group, ^#^P < 0.05 compared with the CFA group, two-way analysis of variance followed by Fisher’s least significant difference post hoc test).

Figures 6 and 9 depict the CGRP expression in bilateral TNCs in all groups. Similar to that in TG, CFA injection immediately and significantly enhanced the expression of CGRP in the ipsilateral TNC at post–CFA-injection days 3, 7, and 14, but not in the contralateral TNC. Hence, CFA-induced inflammation on the ipsilateral side immediately activated the trigeminal pain pathway, but not on the contralateral side. Although 30 and 300 mg/kg ECa 233 could not reduce the enhanced expression of CGRP in the ipsilateral TNC, ibuprofen and 100 mg/kg ECa 233 effectively reduced the enhanced CGRP expression compared with the CFA injection. Therefore, 100 mg/kg ECa 233 effectively inhibited the inflammation in TNC.

#### Expression of p-CREB in TNC

**Figure 7:**
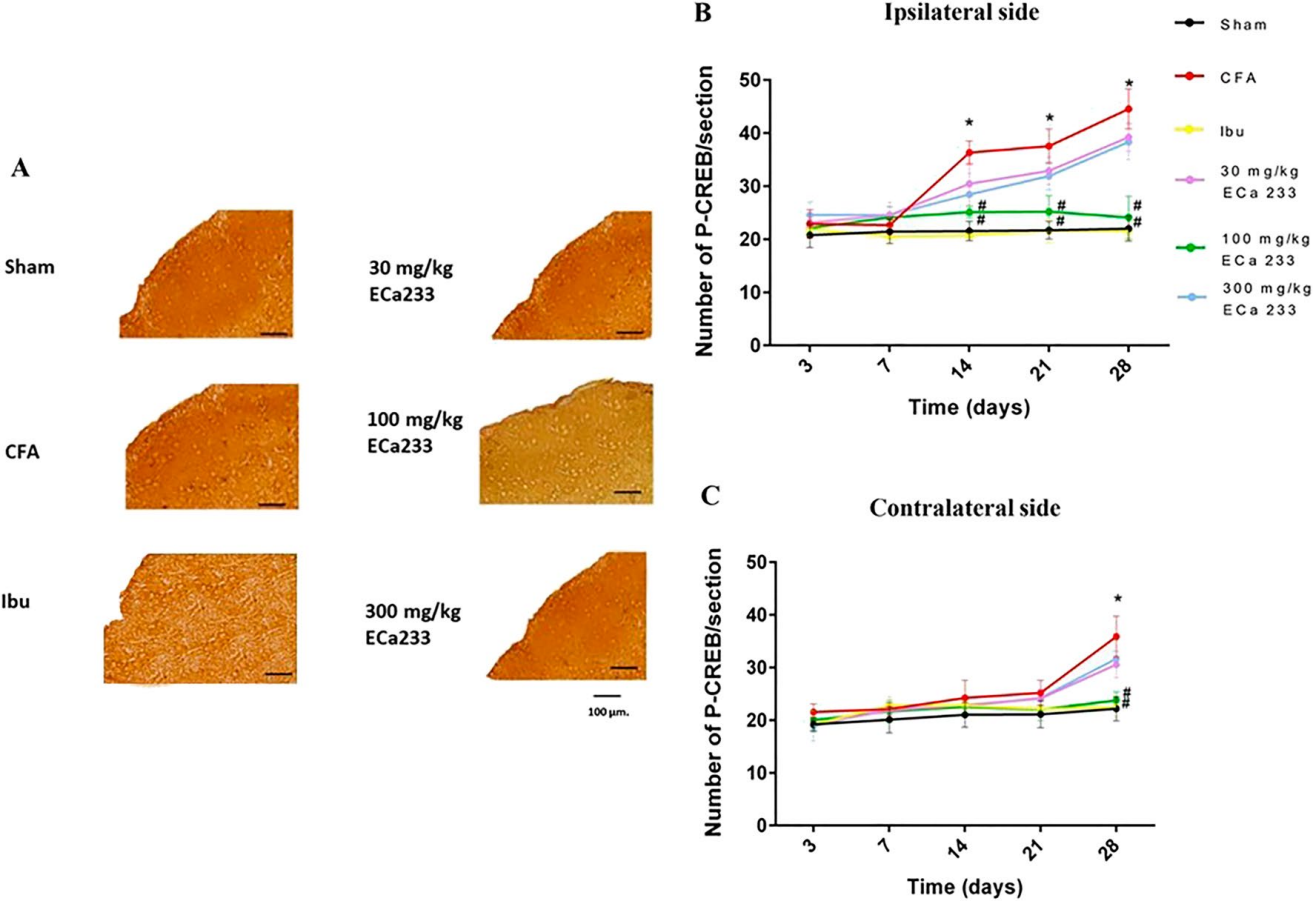
Phosphorylated cAMP response element-binding protein (p-CREB) expression in trigeminal subnucleus caudalis (TNC) in all groups at different time points. (**A**) An example of p-CREB staining by immunohistochemistry at the ipsilateral TNC at post–CFA-injection day 28, scale bar = 100 µm. (**B**,**C**) Expression of p-CREB in both sides of TNCs in different groups. In the ipsilateral TNC at post–CFA-injection days 14, 21, and 28 and in the contralateral TNC at post–CFA-injection day 28, the p-CREB expression in the complete Freund’s adjuvant–treated (CFA) group increased compared with that in the Sham group (on the ipsilateral side: day 14 [F(5,48) = 35.179, P < 0.05], day 21 [F(5,48) = 34.179, P < 0.05], day 28 [F(5,48) = 33.789, P < 0.05]; on the contralateral side: day 28 [F(5,48) = 26.885, P < 0.05]), whereas that in the groups treated with ibuprofen and 100 mg/kg ECa 233 (Ibu and 100ECa groups, respectively) significantly decreased compared with that in the CFA group (day 14 on the ipsilateral side: Ibu [F(5,48) = 21.225, P < 0.05], 100ECa [F(5,48) = 25.247, P < 0.05]; day 21 on the ipsilateral side: Ibu [F(5,48) = 22.375, P < 0.05], 100ECa [F(5,48) = 26.027, P < 0.05]; day 28 on the ipsilateral side: Ibu [F(5,48) = 24.335, P < 0.05], 100ECa [F(5,48) = 26.247, P < 0.05]; day 28 on the contralateral side: Ibu [F(5,48) = 26.245, P < 0.05], 100ECa [F(5,48) = 27.161, P < 0.05]). Both 30ECa and 300ECa showed no difference from the CFA group (day 14 on the ipsilateral side: 30ECa [F(5,48) = 40.485, P = 0.207], 300ECa [F(5,48) = 39.532, P = 0.236]; day 21 on the ipsilateral side: 30ECa [F(5,48) = 40.532, P = 0.196], 300ECa [F(5,48) = 39.532, P = 0.179]; day 28 on the ipsilateral side: 30ECa [F(5,48) = 41.485, P = 0.187], 300ECa [F(5,48) = 39.852, P = 0.196]; day 28 on the contralateral side: 30ECa [F(5,48) = 40.495, P = 0.197], 300ECa [F(5,48) = 39.972, P = 0.194]) (mean ± SEM; n = 3/group at each time; *P < 0.05 compared with the Sham group, ^#^P < 0.05 compared with the CFA group, two-way analysis of variance followed by Fisher’s least significant difference post hoc test).

Figures 7 and 9 show the expression of p-CREB in bilateral TNCs in all groups. In the CFA group, the expression of p-CREB significantly increased later than that of CGRP on the ipsilateral side at post–CFA-injection days 14, 21, and 28, and on the contralateral side at post–CFA-injection day 28 compared with that in the Sham group. The enhanced expression of p-CREB on both sides corresponded well with the pain hypersensitivity on each side. Thus, the underlying mechanisms of the bilateral pain hypersensitivity development most likely came from the overstimulation of both TG (in the PNS) and TNC (in the CNS) on the ipsilateral side and of only the TNC on the contralateral side. Although 30 and 300 mg/kg ECa 233 could not reduce the enhanced expression of p-CREB on both sides, ibuprofen and 100 mg/kg ECa 233 effectively abolished the enhanced p-CREB expression on both sides compared with the CFA injection. Thus, 100 mg/kg dose of ECa 233 effectively prevented central sensitization and pain hypersensitivity in TNC.

#### Expression of activated microglia in TNC

**Figure 8:**
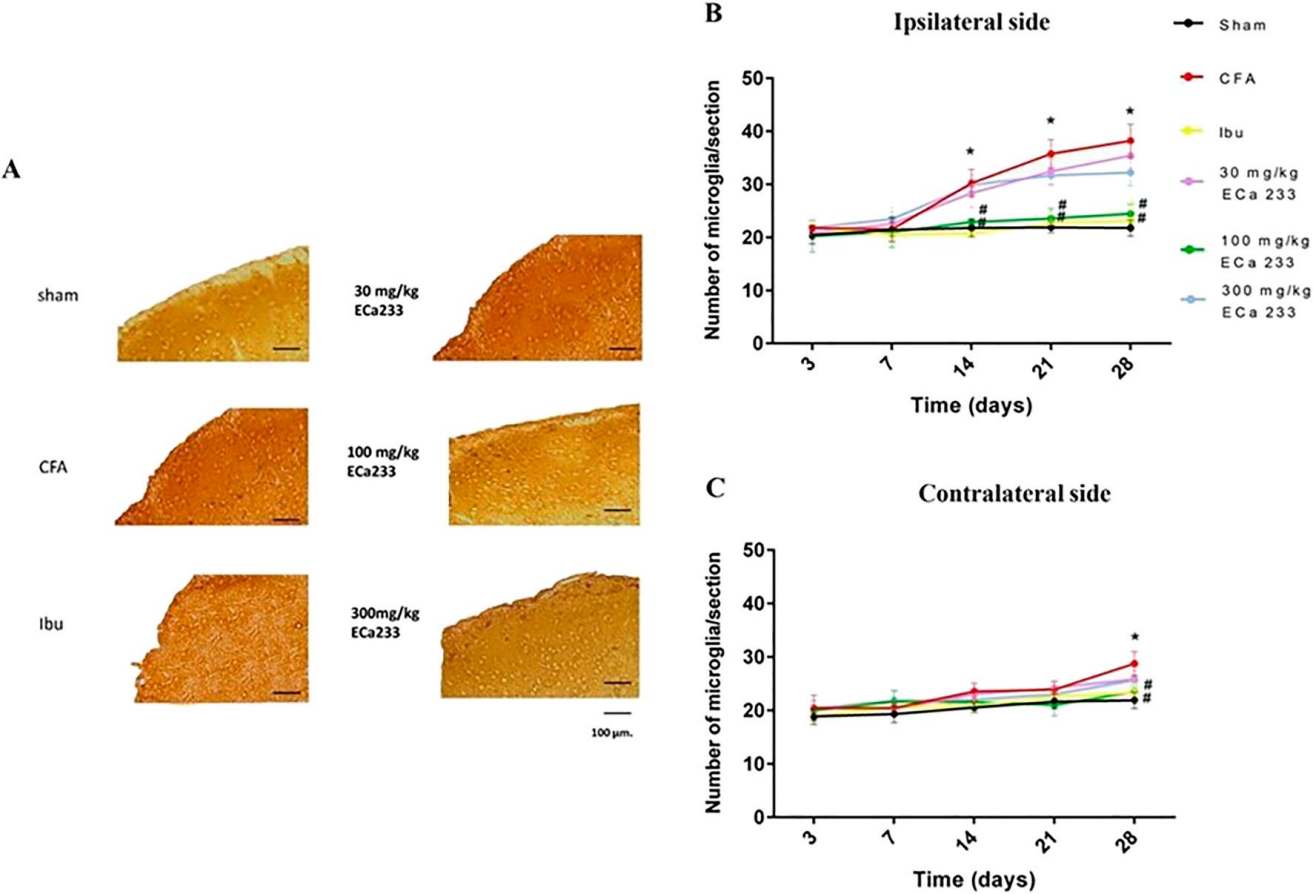
Expression of activated microglia in trigeminal subnucleus caudalis (TNC) in all groups at different time points. (**A**) An example of activated microglia staining by immunohistochemistry at the ipsilateral TNC at post–CFA-injection day 28, scale bar = 100 µm. (**B**,**C**) Expression of activated microglia on both sides of TNC in the different groups. In the ipsilateral TNC at post–CFA-injection days 14, 21, and 28 and in the contralateral TNC at post–CFA-injection day 28, the expression of activated microglia in the complete Freund’s adjuvant–treated (CFA) group increased compared with that in the Sham group (on the ipsilateral side: day 14 [F(5,48) = 32.013, P < 0.05], day 21 [F(5,48) = 29.084, P < 0.05], day 28 [F(5,48) = 34.027, P < 0.05]; on the contralateral side: day 28 [F(5,53) = 25.521, P < 0.05]), whereas that in the groups treated with ibuprofen and 100 mg/kg ECa 233 (Ibu and 100ECa groups, respectively) significantly decreased compared with that in the CFA group (day 14 on the ipsilateral side: Ibu [F(5,48) = 20.135, P < 0.05], 100ECa [F(5,48) = 22.154, P < 0.05]; day 21 on the ipsilateral side: Ibu [F(5,48) = 22.500, P < 0.05], 100ECa [F(5,48) = 25.034, P < 0.05]; day 28 on the ipsilateral side: Ibu [F(5,48) = 24.335, P < 0.05], 100ECa [F(5,48) = 26.154, P < 0.05]; day 28 on the contralateral side: Ibu [F(5,48) = 27.135, P < 0.05], 100ECa [F(5,48) = 27.247, P < 0.05]). Both 30ECa and 300ECa showed no difference from the CFA group (day 14 on the ipsilateral side: 30ECa [F(5,48) = 35.545, P = 0.315], 300ECa [F(5,48) = 36.005, P = 0.309]; day 21 on the ipsilateral side: 30ECa [F(5,48) = 35.785, P = 0.335], 300ECa [F(5,48) = 40.005, P = 0.319]; day 28 on the ipsilateral side: 30ECa [F(5,48) = 39.785, P = 0.375], 300ECa [F(5,48) = 41.305, P = 0.329]; day 28 on the contralateral side: 30ECa [F(5,48) = 39.894, P = 0.385], 300ECa [F(5,48) = 42.385, P = 0.362]) (mean ± SEM; n = 3/group/time point; *P < 0.05 compared with the Sham group, ^#^P < 0.05 compared with the CFA group, two-way analysis of variance followed by Fisher’s least significant difference post hoc test).

**Figure 9:**
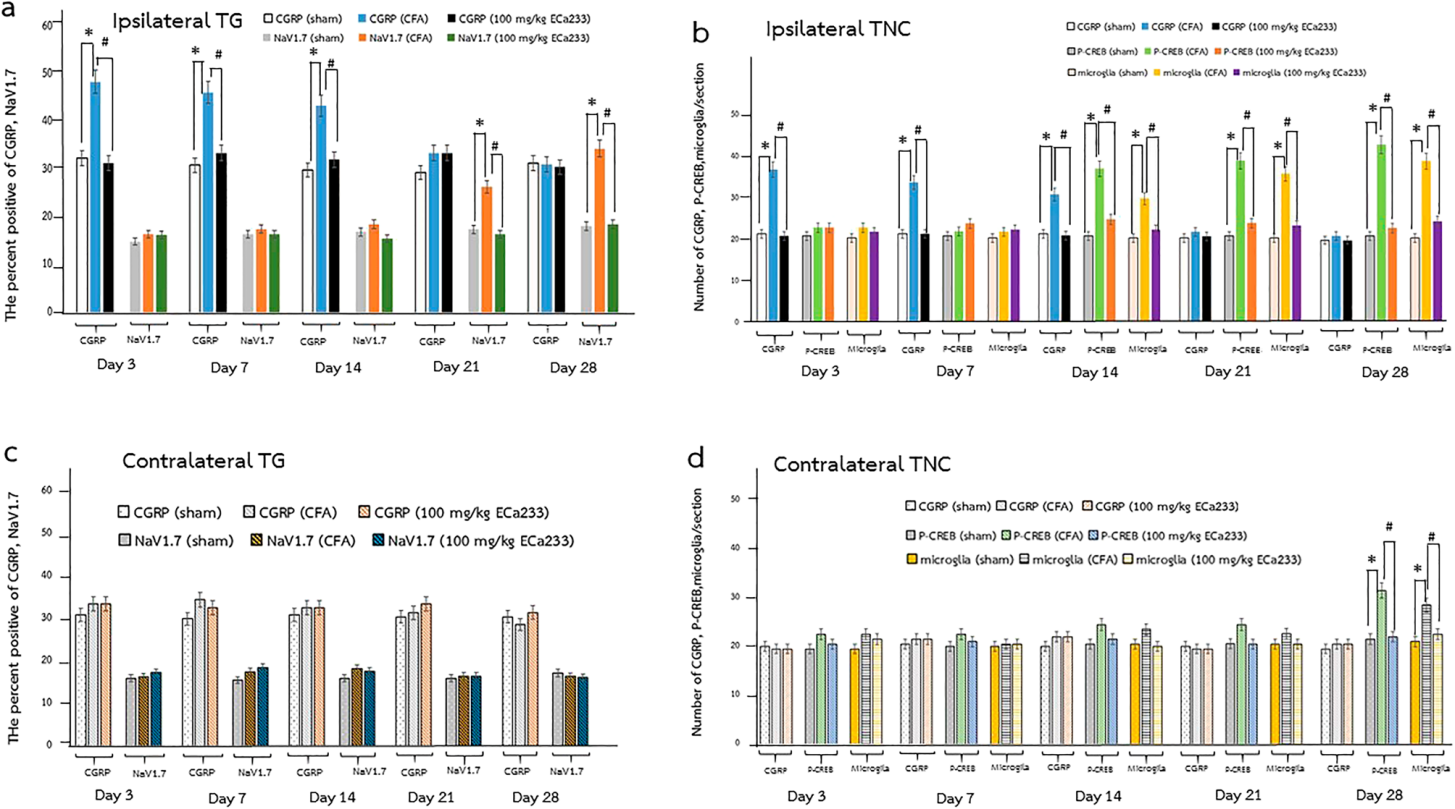
Expressions of CGRP and NaV1.7 in TG, and expressions of CGRP, p-CREB, and activated microglia in TNC in Sham, CFA, and 100ECa groups at different time points. (**a**) Expressions of CGRP and NaV1.7 in the ipsilateral TG. (**b**) Expressions of CGRP, p-CREB, and activated microglia in the ipsilateral TNC. (**c**) Expressions of CGRP and NaV1.7 in the contralateral TG. (**d**) Expressions of CGRP, p-CREB, and activated microglia in the contralateral TNC. (mean ± SEM; n = 3/group/time point; *P < 0.05 compared with the Sham group, ^#^P < 0.05 compared with the CFA group, two-way analysis of variance followed by Fisher’s least significant difference post hoc test).

Figures 8 and 9 present the expression of activated microglia in bilateral TNCs in all groups. In the CFA group, the expression of activated microglia in TNC was later significantly increased on the ipsilateral side at post–CFA-injection days 14, 21, and 28, and on the contralateral side at post–CFA-injection day 28 compared with that in the Sham group. The increase in the expression of the activated microglia in TNC was similar to that in the expression of p-CREB in TNC; hence, CFA-induced inflammation activated the ipsilateral pain pathway. However, on the contralateral side, the p-CREB expression increased, but the CGRP expression did not increase; thus, the pain signals triggering both the contralateral TNC neurons and microglia possibly came from the ipsilateral TNC neurons or higher relay stations. Although 30 and 300 mg/kg ECa 233 could not reduce the expression of activated microglia on both sides, ibuprofen and 100 mg/kg ECa 233 effectively abolished the enhanced expression of activated microglia on both sides compared with the CFA injection. Therefore, 100 mg/kg dose of ECa 233 effectively inhibited the inflammation in TNC.

## Discussion

This is the first in vivo study of the standardized extract of *C. asiatica* (ECa 233) reporting that ECa 233 could attenuate pain hypersensitivity in chronic inflammatory TMD in male mice induced by CFA injection. Despite inducing many side effects, such as gastrointestinal irritation and kidney injury, the NSAID ibuprofen is currently the standard treatment for TMD pain^15^.

After receiving ibuprofen and ECa 233 (30 and 100 mg/kg), the mice had lower pain response scores. However, only ibuprofen and 100 mg/kg ECa 233 could protect the TMJ from bone density changes and prevent the increased expression of inflammation- and nociception-associated proteins in the TG (PNS relay station) and TNC (CNS relay station) of the trigeminal pain pathway. Although the pathophysiology of chronic inflammatory TMD pain remains unclear, other studies hypothesized that TMD results from the local release of inflammatory irritants caused by tissue damage or external irritants such as CFA. Clinically, however, many patients with TMD have no apparent signs of local inflammation or tissue damage^16^, and TMD pain can widely spread within the orofacial area^17^.

In our TMD model, CFA caused an excessive uncontrolled inflammation mimicking the osteoarthritis-induced TMD pain^18^. Owing to the inflammation at the injected TMJ, the TMJ’s condylar head degenerated with lowered bone density, leading to osteoarthritis-induced pain hypersensitivity^19^. Consistent with the previous studies, we showed that the unilateral CFA injection induced chronic inflammation in the ipsilateral joint and nervous system (both PNS and CNS); hence, hyperalgesia and allodynia occur on the ipsilateral side. However, through our lengthened observation into the chronic phase of pain (28 days after the CFA injection), our study also demonstrated changes that occurred in the contralateral CNS, causing pain hypersensitivity on the contralateral side, as first reported by our group recently, indicative of a mirror-image pain^20^.

When stimulated by noxious stimuli, CGRP drives the sensitization at TG and TNC in the chronic phase of the TMD pain, as evidenced by the increased expression of NaV1.7 in TG and that of p-CREB in TNC. In a previous study, injecting CGRP into the rat’s TMJ promoted changes in p38 MAP kinases and ERK in TG and c-Fos in TNC after 2 h of injection, indicating peripheral and central sensitizations, respectively^21^. Meanwhile, pain hypersensitivity and neuronal activity in the trigeminal system in response to CFA-induced pain in the masseter muscle were attenuated by a CGRP antagonist^22^. In our study, CGRP was enhanced only in the early stage of inflammation in the ipsilateral TG and TNC, but not in the contralateral TG and TNC; thus, the pain signals stimulating the contralateral TNC (as evidenced by the late enhancement of the expressions of p-CREB and activated microglia at post–CFA-injection day 28) would originate from the ipsilateral TNC or other higher relay stations, eventually producing pain hypersensitization at post–CFA-injection day 28 on the contralateral side. Therefore, our data supported that TMD pain could occur despite the lack of inflammatory signs as in the mirror-image pain.

In particular, NaV had implicated chronic pain including NaV1.7, NaV1.8, NaV1.9. However, in terms of insight into function, NaV1.7, which is related to communication in nociceptive peripheral afferents, has a hereditary loss-of-function mutation that triggers pain hypersensitivity without causing other neurodevelopmental changes^23^. In an animal model, successful NaV1.7 suppression in the lumbar dorsal root ganglia was associated with decreased thermal hyperalgesia in the inflammatory state, decreased tactile allodynia in the neuropathic state, and no modifications to the animals' regular motor function^23,24^. Therefore, in our study the increased expression of NaV1.7, a pain hypersensitivity marker in TG, would lower stimulation threshold and provoke ectopic discharge, causing peripheral sensitization^3^. Moreover, activation of p-CREB, a molecular marker of pain transmission and pain hypersensitivity in TNC^4^, may be a key step for developing a synaptic plasticity–dependent activity that ultimately increases pain perception^25^. The role of activated microglia in TNC and spinal cord is related to pain hypersensitivity, particularly mechanical hyperalgesia following inflammation^26^. Persistent PNS inflammation is a major cause of activated microglia in CNS. Activated microglia release proinflammatory cytokines and chemokines, leading to widespread inflammatory responses. Our study revealed expressions of NaV1.7, p-CREB, and activated microglia in the 100ECa and Ibu groups significantly decreased compared with that in the CFA group. Thus, the 100-mg/kg dose of ECa 233 could prevent pain sensitization, possibly by reducing the CGRP and NaV1.7 expressions, thereby reducing the behavioral pain responses and pain hypersensitivity.

Consistent with other supporting results, *C. asiatica* extracts and active ingredients had shown both anti-inflammatory and antinociceptive effects. For example, 100 mg/kg dose of *C. asiatica* inhibited foot edema in carrageenan and prostaglandin-induced hind foot pain in rats, similar to the activity of ibuprofen treatment^8^. *C. asiatica* also significantly decreased proinflammatory cytokine (TNF-α and interleukin 6) expression in lipopolysaccharide-induced HEK-293 cell inflammation^27^. Moreover, oral madecassoside administration could reduce the clinical pain scores of mice with an induced rheumatoid arthritis and also minimize prostaglandin, COX-2, and proinflammatory cytokine infiltrations^27^. Indeed, madecassoside promotes anti-inflammation and protects the joint from destruction. Moreover, asiaticoside has an antinociceptive effect, seen when oral treatment of asiaticoside reduced pain in mice with capsaicin-induced paw pain^28^. Therefore, ECa 233, which contains ≥ 80% w/w of triterpenoid glycosides with a 1.5:1 ratio of madecassoside and asiaticoside, possesses both anti-inflammatory and antinociceptive activities, resulting in the reduction of inflammatory responses in both PNS and CNS and behavioral pain responses; however, the effective dose of both activities demonstrated an inverted U-shaped dose–response relationship with the maximal effect at 100 mg/kg dose.

At a low dose of 30 mg/kg, ECa 233 showed an effect comparable with that of 100 mg/kg ECa 233 on pain hypersensitivity alleviation, but it did not reduce the expression of inflammation-associated proteins; thus, the antinociceptive activity of ECa 233 started to manifest at the low dose. The 30 mg/kg dose of ECa 233 contains approximately 15 mg/kg dose of madecassoside and 10 mg/kg dose of asiaticoside^9^. Madecassoside at 40 mg/kg (not 10 and 20 mg/kg) could reduce inflammation, thereby lowering pain scores and protecting the joint from destruction; hence, madecassoside exhibits an anti-inflammatory activity^27^. Meanwhile, asiaticoside at 10 mg/kg (not 5 mg/kg) could alleviate pain in capsaicin-induced pain; hence, asiaticoside has an antinociceptive activity^13^. Therefore, the possible mechanism is that at the dose of 30 mg/kg, ECa 233 could exert the antinociceptive effect, thereby reducing the behavioral pain responses; however, it could not reduce inflammatory responses and prevent osteoarthritis development at the CFA-injected TMJ. ECa 233 at the dose of 100 mg/kg containing approximately 49.5 mg/kg concentration of madecassoside and 33 mg/kg concentration of asiaticoside not only reduced the pain responses but also reduced inflammatory responses and prevented osteoarthritis development.

At the low dose of ECa 233 (30 mg/kg), the antinociceptive activity, which might originate from the asiaticoside activity, was perhaps caused by the increased activity of gamma aminobutyric acid (GABA), which is the major inhibitory neurotransmitter of CNS. The activity of glutamic acid decarboxylase, which is involved in GABA synthesis, is increased by an aqueous extract of *C. Asiatica*^29^. Moreover, asiatic acid, which is an active metabolite of asiaticoside, interacts with GABAB receptor to inhibit synaptic transmission. The increased GABA activity of CNS might cause pain hypersensitivity reduction. However, the GABA expression does not directly relate to the inflammatory markers^30^ and also may not be associated with the expression of inflammation-associated proteins in the pain pathway.

Interestingly, high-dose ECa 233 treatment (300 mg/kg) did not exhibit beneficial effects. One of the reasons could be that the plant products contain other constituents, which at a high dose, exert some antagonistic or inhibitory effects. ECa 233 could enhance brain-derived neurotrophic factor (BDNF) and *N*-methyl-D-aspartate receptor (NMDAR) in the hippocampus, cerebral cortex, and spinal cord^9^. BDNF facilitated pain transmission and contributed to pain hypersensitivity development^31^ via the postsynaptic NMDAR to modulate nociceptive signaling in TNC^32^. After BDNF antagonist injection in mice with formalin-induced pain, pain hypersensitivity decreased^33^. Intrathecal NMDAR antagonist injection in nerve injury-induced neuropathic pain reduced pain hypersensitivity^34^. Therefore, BDNF and NMDAR were involved in some aspects of pain hypersensitivity, possibly in the central sensitization. Thus, at a high dose (300 mg/kg), ECa 233 may facilitate pain transmission, counteracting its antinociceptive and anti-inflammatory effects. All these mechanisms could perhaps inhibit the beneficial effects of the major constituents, possibly owing to which the highest dose of ECa 233 exhibited results similar to those of the control group.

Therefore, the inverted U-shaped dose–response relationship of ECa 233 might indicate one of the factors responsible for pain hypersensitivity reduction. Hence, the effective dose of *C. asiatica* extract should be limited to moderate doses to achieve beneficial effects in chronic inflammatory TMD pain. The recommended dose of ECa 233 in this study is 100 mg/kg, which showed both antinociceptive and anti-inflammatory effects by preventing the anatomical changes in the condylar head of TMJ and hindering the expression of inflammation- and nociception-associated proteins in the TG and TNC. These findings provide basic knowledge of ECa 233 effects on inflammation and nociception, supporting ECa 233 development as an antinociceptive and anti-inflammatory drug or a dietary supplement for pain treatment.

Our study's limitations include not using the rotarod test for evaluating ECa 233 additional behavioral side effects, and consideration of the subjective meaning of pain. Moreover, NaV implicated in pain such as NaV1.8, NaV1.9 in the trigeminal pathway should be further studied.

In conclusion, *C. asiatica* has antinociceptive and anti-inflammatory effects on chronic inflammatory TMD pain within a specific therapeutic range. Despite the very limited therapeutic dose of ECa 233, it has potent effects and a high safety profile. Therefore, ECa 233 can be safely used as an antinociceptive and anti-inflammatory agent in chronic pain management, although further investigation is still required.

## Methods

### Animals

We used 6-week-old healthy male mice (n = 144, weighing 25–30 g, which were purchased from the Nomura Laboratory Animal Center, Thailand. All mice were housed under conditions of controlled humidity (40%–70%), temperature (23 °C ± 1 °C), and light (12 h day/12 h night cycle), with free water and food access. All study protocols were performed in accordance with relevant guidelines and regulations and were approved by the Animal Care and Use Committee of the Faculty of Medicine Siriraj Hospital, Mahidol University (Approval No.: SI-ACUP014/2561). All experimental procedures conformed to the Animal Research: Reporting In Vivo Experiments (ARRIVE) guidelines for animal experiment^35^. The sample size was estimated to provide 80% power (1 − β) with a 95% confidence interval (α = 0.05)^36^. According to previous study, CFA-induced TMJ pain animal model was treated with ibuprofen treatment as a positive control^13,37^ . A total of 144 mice were used for the study, randomly divided into six groups (n = 24/group):

1. Sham (Sham): 10 µl of 0.9% normal saline solution (NSS) injection
2. Negative control (CFA): 10 µl of 50% CFA injection
3. Positive control (Ibu): 10 µl of 50% CFA injection + ibuprofen (0.14 g/kg)
4. 30 mg/kg ECa 233 (30ECa): 10 µl of 50% CFA injection + ECa 233 (30 mg/kg)
5. 100 mg/kg ECa 233 (100ECa): 10 µl of 50% CFA injection + ECa 233 (100 mg/kg)
6. 300 mg/kg ECa 233 (300ECa): 10 µl of 50% CFA injection + ECa 233 (300 mg/kg)

To evaluate pain hypersensitivity, we used 54 animals (9 animals/group) for the behavioral pain response tests at pre–CFA-injection day and post–CFA-injection days 3, 7, 14, 21, and 28. At each time point of post–CFA-injection days 3, 7, 14, 21, and 28, 90 animals (3 animals/group/time point) were used for evaluating the structural changes caused by CFA-induced inflammation in bilateral TMJs by assessing the bone density through micro–computed tomography (micro-CT) and the expression of proteins related to the inflammatory and nociceptive activities in TG (CGRP and NaV1.7) and TNC (CGRP, p-CREB, and activated microglia) through immunohistochemistry.

### Development of TMD

To induce temporomandibular pain, we injected the mice with sodium pentobarbital (60 mg/kg) intraperitoneally for anesthesia and then with 10 µl of NSS-dissolved 50% CFA (1 mg/ml) (F5881; Sigma-Aldrich, St. Louis, MO, USA) unilaterally into the right TMJ. The anatomical landmark for the injection was described in a previous study^20,38^. Briefly, we identified TMJ by palpation and trimmed the surrounding local hair using a pair of scissors. We subsequently inserted a 30-gauge needle through the facial skin until the needle tip reached the zygomatic arch. We slowly advanced the needle until it passed under the edge of the arch and ultimately entered into the joint space. When the needle reached the joint space, we slowly injected 10 µl of 50% CFA, via a Hamilton syringe, over a period of 5 s.^20,38^.

### Behavioral test

We performed two series of behavioral pain response tests [using 0.04 g of von Frey filament for mechanical hyperalgesia and 0.05 ml of cold acetone (− 5 °C) for cold allodynia with an interval of at least 30 min. At pre–CFA-injection day and post–CFA-injection days 3, 7, 14, 21, and 28, the mice underwent tests at their whisker pads of both ipsilateral and contralateral sides. We tested each side 12 times and then recorded the response scores according to the following criteria: no response = 0, occurrence of head withdrawal = 0.25, occurrence of face grooming once = 1, and occurrence of face grooming of > 3 times = 1.5^18,20^. We added all response scores on each side to obtain the total response score; the maximum response score was 18.

### Micro-CT of TMJ

Animals were deeply anesthetized and then transcardially perfused with 250 ml of ice-cold phosphate-buffered solution (PBS; 0.1 M, pH 7.4). The head of the mice was decapitated, immersed in 4% paraformaldehyde in PBS (0.1 M, pH 7.4), and placed horizontally in a micro-CT scan (µCT 35 Sanco Medical AG, Bruetteisellen, Switzerland). We scanned the condylar head of TMJ using an X-ray at 30–70 kVp. Thereafter, we sliced the condylar head of TMJ and selected the slices from the start of the condylar head to the neck of the ramus. Normally, approximately 150–250 sections or slices are obtained^39^. The condylar head of TMJ was analyzed by measuring the bone density of each mouse in every group at different time points (post–CFA-injection days 3, 7, 14, 21, and 28).

### Inflammatory and nociceptive activities in TG and TNC

The mice were deeply anesthetized and perfused with the same conditions used for the micro-CT. We located TG and TNC using the brain atlas of Paxinos and Keith^40^. TG and TNC were then extracted bilaterally and immersed in 4% paraformaldehyde in PBS (0.1 M, pH 7.4) immediately. Tissue processing and paraffin embedding procedures were also performed. We transversely sliced 5-μm thick sections using a microtome (Global Medical Instrumentation Inc., Ramsey, MN, USA) and deparaffinized them before immunostaining by immunohistochemistry. The TG paraffin sections were stained with neuroinflammatory markers rabbit monoclonal anti-NaV1.7 antibody (AB5390; Merck KGaA, Darmstadt, Germany) and rabbit polyclonal anti-CGRP antibody (SC-57053; Santa Cruz Biotech, Dallas, TX, USA), whereas the TNC sections were immunolabeled with a rabbit polyclonal anti-p-CREB antibody (SC-81486; Santa Cruz Biotech, Dallas, TX, USA), rabbit polyclonal anti-CGRP antibody (SC-57053; Santa Cruz Biotech, Dallas, TX, USA), and rabbit polyclonal anti-OX42 antibody (SC-52; Santa Cruz Biotech, Dallas, TX, USA) for the expression of activated microglia.

Briefly, the TG and TNC sections were warmed with antigen retrieval solution (citrate buffer; Dako, Glostrup, Denmark) in a microwave oven. The endogenous peroxidase activity was inhibited with Dako Peroxidase Blocking Reagent (Dako) for 5 min and then blocked with an antibody diluent (Dako) for 10 min to prevent nonspecific staining. Further, we incubated the sections with the primary antibodies at 4 °C overnight, followed by the secondary antibody (goat anti-rabbit-IgG antibody conjugated with horseradish peroxidase). We used the EnVision™ Detection System (Dako) for signal visualization and a light microscope (Carl Zeiss Microimaging GmbH, AxioVision 40 version 4.8.2.0, Germany) for peroxidase activity site detection. We also evaluated the nociceptive activity in TG and TNC individually and independently and blindly analyzed each datum by the mean number of positive neuron expression at each time point.

For TG analysis, we used three mice from each group at each time point and selected five sections per TG tissue (5th, 10th, 15th, 20th, and 25th sections) of each mouse. We counted the neurons in each whole TG section and then summed and averaged the numbers of counted neurons from all sections in one mouse. The ratio of positive neurons per total number of neurons and the mean value of each experimental group (n = 3/group/time point) were calculated. For TNC analysis, we used the same three mice from each group at every time point and selected five sections per TNC tissue (5th, 10th, 15th, 20th, and 25th sections) of each mouse. We counted the positive cells (brown dots) at laminas I and II of the TNC sections and then summed and averaged the numbers of brown dots from all sections in one mouse. Thereafter, the mean value of each experimental group (n = 3/group/time point) was analyzed.

### Statistical analysis

All statistical data were analyzed using SPSS version 26.0 (IBM, Chicago, IL, USA). The Kolmogorov–Smirnov test was used for testing normal data distribution. Possible statistically significant differences between groups were determined using two-way analysis of variance with Fisher’s least significant difference post hoc test. All analyses were performed using the two-tailed hypothesis testing method. In addition, P < 0.05 was considered statistically significant. Data are shown as mean ± standard error of the mean.

## Data Availability

The datasets generated and analyzed during the current study are available from the corresponding author on a reasonable request.
