# Author Correction: Standardized *Centella asiatica* (ECa 233) extract decreased pain hypersensitivity development in a male mouse model of chronic inflammatory temporomandibular disorder

**DOI:** 10.1038/s41598-023-39016-6

**Published:** 2023-07-21

**Authors:** Nattapon Rotpenpian, Aree Wanasuntronwong, Sompol Tapechum, Anchalee Vattarakorn, Chit Care, Wongsathit Chindasri, Kanokwan Tilokskulchai, Mayuree H. Tantisira, Narawut Pakaprot

**Affiliations:** 1 Department of Physiology, Faculty of Medicine Siriraj Hospital, Mahidol University, 2 Srisavarindhira Bldg., 13Th Floor, Wanglang Road, Siriraj Subdistrict, Bangkoknoi District, Bangkok, 10700 Thailand; 2 Department of Oral Biology and Occlusion, Faculty of Dentistry, Prince of Songkla University, Songkhla, Thailand; 3 Department of Oral Biology, Faculty of Dentistry, Mahidol University, Bangkok, Thailand; 4 Faculty of Pharmaceutical Sciences, Burapha University, Chonburi, Thailand

Correction to: *Scientific Reports* 10.1038/s41598-023-33769-w, published online 24 April 2023

The original version of this Article contained errors in the names of the authors Nattapon Rotpenpian, Aree Wanasuntronwong, Sompol Tapechum, Anchalee Vattarakorn, Chit Care, Wongsathit Chindasri, Kanokwan Tilokskulchai, Mayuree H. Tantisira & Narawut Pakaprot, which were incorrectly given as Rotpenpian Nattapon, Wanasuntronwong Aree, Tapechum Sompol, Vattarakorn Anchalee, Care Chit, Chindasri Wongsathit, Tilokskulchai Kanokwan, Tantisira H. Mayuree & Pakaprot Narawut.

The original Article has been corrected.

